# Emerging Roles of Epigenetics in Grapevine and Winegrowing

**DOI:** 10.3390/plants13040515

**Published:** 2024-02-13

**Authors:** Xenophon Venios, Danai Gkizi, Aspasia Nisiotou, Elias Korkas, Sotirios E. Tjamos, Christos Zamioudis, Georgios Banilas

**Affiliations:** 1 Department of Wine, Vine and Beverage Sciences, University of West Attica, Ag. Spyridonos 28, 12243 Athens, Greece; xvenios@uniwa.gr (X.V.); dgkizi@uniwa.gr (D.G.); elkorkas@uniwa.gr (E.K.); 2 Institute of Technology of Agricultural Products, Hellenic Agricultural Organization “Demeter”, Sofokli Venizelou 1, 14123 Lykovryssi, Greece; anisiotou.wi@nagref.gr; 3 Laboratory of Plant Pathology, Agricultural University of Athens, 75 Iera Odos Str., 11855 Athens, Greece; sotiris@aua.gr; 4 Department of Agricultural Development, Democritus University of Thrace, Pantazidou 193, 68200 Orestiada, Greece; czamioud@agro.duth.gr

**Keywords:** epigenomics, climate change, stress responses, plant defense, phenotypic plasticity, viticulture, berry ripening, wine

## Abstract

Epigenetics refers to dynamic chemical modifications to the genome that can perpetuate gene activity without changes in the DNA sequence. Epigenetic mechanisms play important roles in growth and development. They may also drive plant adaptation to adverse environmental conditions by buffering environmental variation. Grapevine is an important perennial fruit crop cultivated worldwide, but mostly in temperate zones with hot and dry summers. The decrease in rainfall and the rise in temperature due to climate change, along with the expansion of pests and diseases, constitute serious threats to the sustainability of winegrowing. Ongoing research shows that epigenetic modifications are key regulators of important grapevine developmental processes, including berry growth and ripening. Variations in epigenetic modifications driven by genotype–environment interplay may also lead to novel phenotypes in response to environmental cues, a phenomenon called phenotypic plasticity. Here, we summarize the recent advances in the emerging field of grapevine epigenetics. We primarily highlight the impact of epigenetics to grapevine stress responses and acquisition of stress tolerance. We further discuss how epigenetics may affect winegrowing and also shape the quality of wine.

## 1. Introduction

Grapevine (*Vitis vinifera* L.) is the most important perennial fruit crop in the world, mainly for wine production, but also for table grapes and raisins [1]. The global wine industry, which heavily relies on grapes, contributes significantly to the economy of many countries. Vines have a long history of cultivation, dating back thousands of years. Although the ancient cultivation of grapevine was mostly restricted in the eastern Mediterranean basin, domesticated germplasm gradually diffused from east to west, on the opposite shores of the Mediterranean [2]. Nowadays, there are about 85 wine-producing countries in the world, while the global vineyard area accounts for about 7.3 million hectares (www.oiv.int/sites/default/files/documents/OIV_Annual_Assessment-2023.pdf, accessed on 1 December 2023). The high geographical dispersal of grapevines in different climatic and edaphic conditions around the world implies its strong ability to adapt to diverse environments. Nevertheless, new challenges arise due to climate change, including its impact on global warming, water availability, and the expansion of pests and diseases. Thus, the knowledge of grapevine responses to biotic and abiotic stresses is of particular importance for a sustainable viticulture. 

Significant research, based on classical physiology combined with cellular and molecular approaches, has been conducted to better understand responses and regulatory elements that control grapevine acclimation and adaptation to unfavorable environmental conditions [3,4,5,6]. Based on field observations, grapevines exhibit a remarkable ability to adopt to environmental fluctuations, a phenomenon called phenotypic plasticity [7]. Phenotypic plasticity is crucial for grapevine cultivation worldwide, as it allows an organism to exhibit different phenotypes in response to environmental changes without alterations in its underlying DNA sequence [8]. Phenotypic plasticity is often mediated by epigenetic modifications. The term “epigenetics” refers to the molecular procedures that regulate gene expression without altering the underlying DNA sequence. In multicellular organisms, epigenetic mechanisms are vital for the development, differentiation, and maintenance of different cell types. Moreover, epigenetics plays a critical role in plant adaptation to unfavorable conditions by providing a mechanism for heritable and reversible changes in gene expression patterns. Environmental stresses such as drought, extreme temperatures, and pathogens can induce epigenetic alterations in plants, which instigate variation in gene expression and enable plant adaptive responses [9].

During epigenetic regulation, small chemical groups, such as methyl or acetyl groups, are added on or removed from the double helix of the DNA or the associated histones. In a simplistic explanation, these modifications affect how strongly the DNA is bound on histones and, therefore, how easily it can be unraveled for the initiation of transcription [10]. Epigenetic modifications can be potentially inherited by the next generation, giving rise to new phenotypes that cannot be explained by Mendelian genetics [11]. During the last years, accumulating data suggest a prominent role of epigenetics in defense signaling, stress priming, and memory. Epigenetic memory refers to the process by which an external stimulus, either chemical, biotic, or abiotic stressor, induces changes in the epigenetic landscape of an organism that lead to the establishment of stress memory. Upon subsequent such stress events, plants respond more rapidly and effectively [12,13,14]. 

Research on grapevine epigenetics is still in its infancy [15]. Nonetheless, recent studies have indicated that environmental signals can cause epigenetic modifications that impact grapevine adaptation to stress. Furthermore, a number of epigenetically regulated genes involved in grape berry development and ripening have been identified that may also impact grape must composition and thus shape wine characteristics and quality. Here, we provide an overview on the epigenetic mechanisms that have been identified in grapevine and how they may influence responses to environmental stress, disease resistance, and important traits, such as berry ripening and secondary metabolite accumulation in grapes.

## 2. Overview of Epigenetic Mechanisms in Plants

The main epigenetic mechanisms in plants include DNA methylation, histone modifications, and non-coding RNAs (ncRNAs). These mechanisms regulate gene expression by inducing two different gene silencing phenomena, namely, transcriptional gene silencing (TGS), which represses transcription [16], and post-transcriptional gene silencing (PTGS), which involves specific mRNA sequence degradation [17]. 

### 2.1. DNA Methylation

DNA methylation is the most studied epigenetic mechanism in plants, which regulates gene expression by altering the formation of chromatin structure, recruiting gene silencing proteins, and preventing the binding of transcription factors to DNA [18]. It also has an essential role in maintaining DNA stability by suppressing the activity of transposable elements (TEs) and the transcription of potentially harmful exogenous genetic elements (e.g., viral DNA) [19,20]. DNA methylation primarily takes place when a methyl group (-CH3) is covalently attached to the fifth carbon of the pyrimidine ring of cytosine forming 5-methylcytosine (5-mC) or, more rarely, to the sixth carbon of the purine ring of adenine forming N6-methyladenine (N6-mA) [15,21]. 

**Figure 1:**
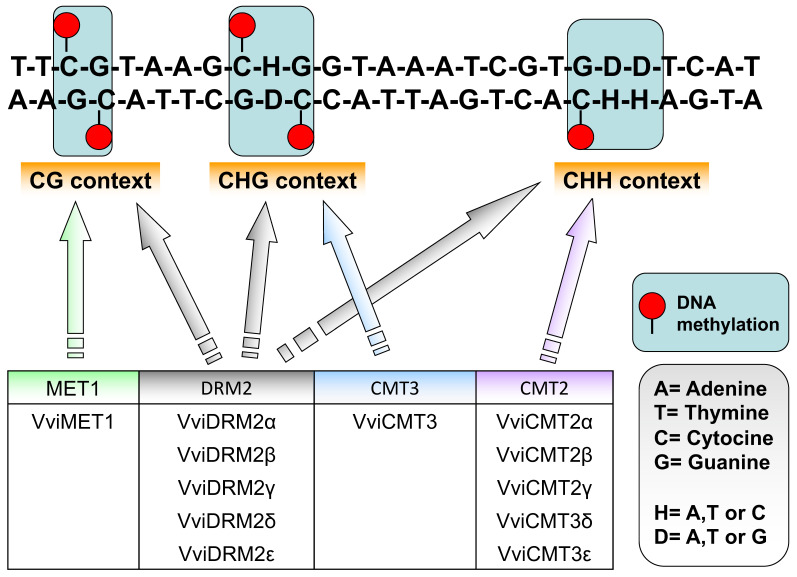
DNA methylation sequence context and DNA methytransferases (DNTMs) of *Vitis vinifera*. MET1 maintains methylation at the CG context, CMT2 and CMT3 at the CHH and CHG contexts, respectively, while DRM2 catalyzes the de novo methylation at all contexts.

In plants, de novo DNA methylation is driven by non-coding RNAs (ncRNAs), which add methyl groups at specific DNA sequences resulting in transcriptional repression through a process called RNA-directed DNA methylation (RdDM) [22]. This process is catalyzed by specific enzymes called DNA methyltransferases (DNMTs), which are responsible for the transfer of methyl groups from the S-adenosyl-l-methionine methyl donor (SAM) to DNA target sites in three different sequence patterns, namely, the symmetricals CG and CHG and the non-symmetrical CHH (where H=Adenine, Thymine, or Cytosine) [9]. Maintenance of cytosine methylation is also carried out by DNMTs and is crucial since its absence invariably results in the passive removal of transcriptional repression after each replication cycle [23]. The maintenance of methylation in CG and CHG sequence contexts is undertaken by methyltransferase 1 (MET1) and chromomethylase 3 (CMT3), respectively, which copy the methylated sequences to the newly synthesized DNA strand. In the CHH sequence context, which cannot be copied between strands, this role is assumed by domains rearranged methyltransferase 2 (DRM2) (via the RdDM pathway) and chromomethylase 2 (CMT2) that remethylate cytosines at each cellular generation [24]. DNA methylation sequence context and nine related DNTMs have been recently identified in grapevine (Figure 1). These grapevine *DNTMs* code for seventeen proteins due to alternative splicing [25]. *MET* genes undergoing alternative splicing have also been reported in rice [26]. Similar numbers of *DNTM* homologues have been identified in Arabidopsis [27], *Oryza sativa* [28], and *Zea mays* [29].

**Figure 2:**
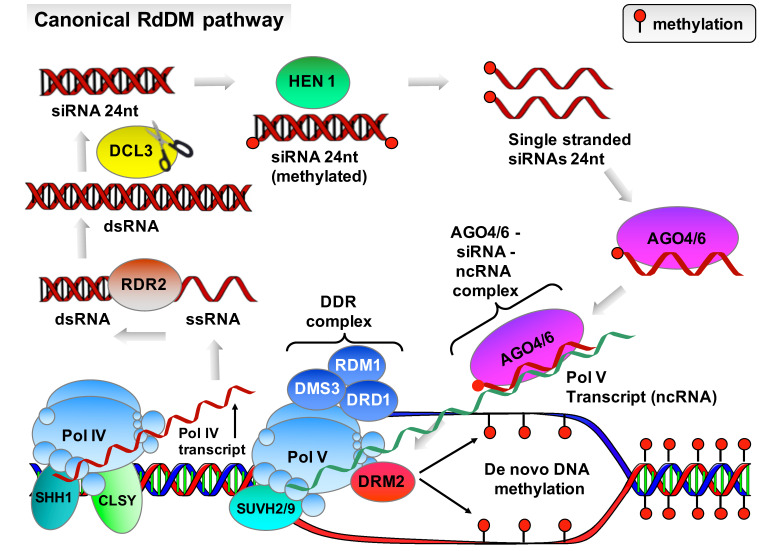
Schematic representation of the canonical RNA-directed DNA methylation (RdDM) pathway.

RdDM involves canonical and non-canonical pathways. These pathways are functionally similar but not identical. During the first stage of the canonical RdDM pathway, the plant specific RNA polymerase IV (Pol IV) complex is recruited to chromatin regions through its interaction with Sawadee homeodomain homolog1 (SHH1) and CLASSY family proteins (CLSYs) [30]. Next, Pol IV transcribes these regions producing short single-stranded RNAs (ssRNAs), which are then converted into double-stranded RNAs (dsRNAs) by the RNA-directed RNA polymerase 2 (RDR2) [31]. These dsRNAs are cleaved by the endoribonuclease enzyme DCL3 into 24-nucleotide small interfering RNAs (siRNAs) and are loaded onto Argonaute 4 (AGO4) or AGO6 proteins forming an AGO–siRNA duplex that enables AGO proteins to recognize and bind to RNA sequences complementary to the siRNA partner [32]. The second stage of this pathway involves the recruitment of Pol V to chromatin by DNA methyl readers SUVH2 and SUVH9 together with the DDR complex (consisting of DMS3, DRD1, and RDM1), leading to the synthesis of Pol V non-coding transcripts [33,34]. These RNA transcripts are used as “scaffolds” onto which the siRNAs loaded onto AGO4 or AGO6 bind forming an AGO–siRNA–ncRNA–Pol V ribonucleoprotein complex. The formation of this complex triggers the recruitment of the DNA methyltransferase domains rearranged methyltransferase 2 (DRM2), which is responsible for targeting the de novo methylation of nearby DNA at all sequence contexts resulting in transcriptional gene silence (Figure 2).

**Figure 3:**
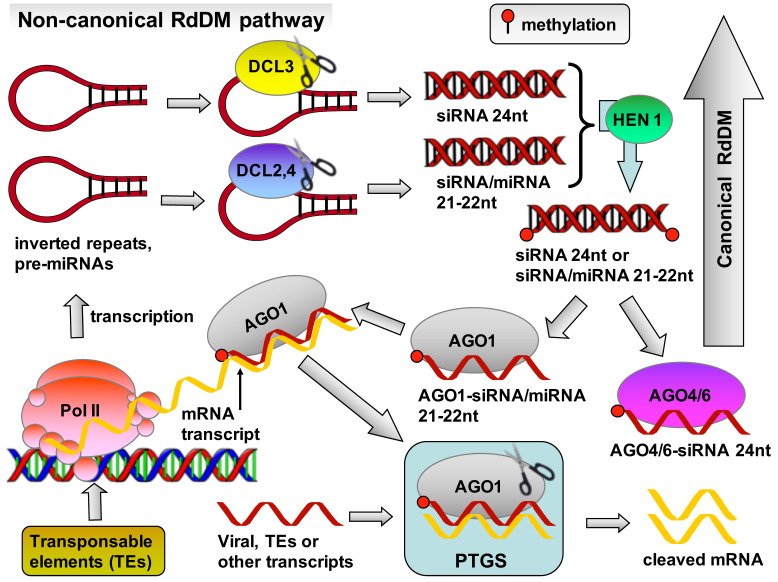
Schematic representation of the non-canonical (or Pol II-dependent) RNA-directed DNA methylation (RdDM) pathway, which forms a bridge connecting the canonical RdDM pathway with post-transcriptional gene silencing (PTGS).

The non-canonical RdDM pathway is generally involved in the initial establishment of DNA methylation at new target loci, such as new transposable element insertions, rather than maintaining the existing silent heterochromatin state [30]. In fact, the non-canonical RdDM pathway often acts as a link between the initial post-transcriptional silencing (PTGS) and the long-term transcriptional silencing (TGS) through the canonical RdDM pathway [35]. The main difference between the two pathways lies in the origin and the production of small RNAs (sRNAs), either siRNAs or miRNAs, involved. Specifically, in contrast to the canonical RdDM pathway that involves 24 nt siRNAs originating solely from Pol IV transcripts, the non-canonical RdDM pathway involves 21–22 nt sRNAs originating from a variety of sources [30]. These 21–22 nt sRNAs are involved not only in the non-canonical RdDM but also participate in other PTGS pathways. Primary sources of these 21–22 nt siRNAs are Pol II transcripts, some of which are directed to PTGS, while others consisting of inverted repeats and miRNA precursors that form double-helix hairpin structures [36]. These hairpin structures can be cleaved by dicer-like proteins (DCL1, 2, 3, or 4) to produce either 21–22 nt sRNAs that bind to AGO1 and participate in PTGS or 24 nt siRNAs that bind to AGO4 or AGO6 and participate in the canonical RdDM pathway (Figure 3).

### 2.2. Histones Post-Translational Modifications (HPTMs)

**Figure 4:**
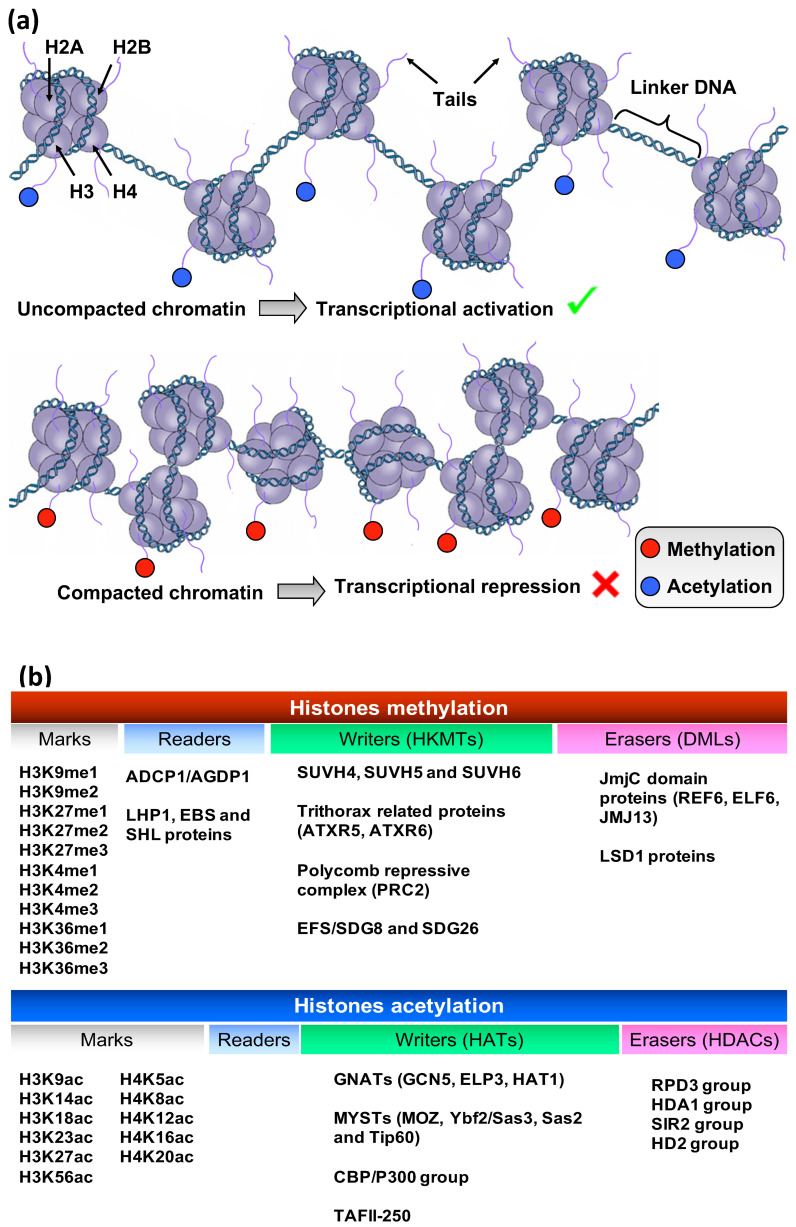
(**a**) Histone H3 methylation and acetylation leading to chromatin structure changes. (**b**) List of important histone marks in plants with their corresponding readers, writers, and erasers.

The structure of nucleosome core particle includes 146 DNA base pairs wrapped around a histone octamer, which consists of two copies from each of the four histone core proteins H2A, H2B, H3, and H4. Additionally, a histone protein, known as H1, binds to the nucleosome core and functions as a linker that stabilizes the structure [37]. These histone core proteins (H2A, H2B, H3, and H4) can be modified by epigenetic enzymes called “writers”, like histone lysine methyltransferases (HKMTs) and acetyltransferases (HATs), which add methyl and acetyl groups, respectively, at the N-terminal of their tails, leading to covalent modifications, i.e., methylation and acetylation [38]. In a similar manner, epigenetic enzymes, like histone demethylases (HDMs) and deacetylases (HDACs), act as “erasers” by removing methyl and acetyl groups, respectively, with the contribution of specific histone “readers” that can detect the position of modifications [39]. In *V*. *vinifera*, in silico analysis revealed the presence of 7 genes coding for HATs and 13 genes for HDACs. Of the seven HATs, two are part of the CBP family (HAC), one is part of the TAFII250 family (HAF), and four are part of the GNAT/MYST family (HAG). Ten out of the thirteen HDACs are members of the RPD3/HDA1 superfamily (HDA), two are members of the SIR2 family (SRT sequences), and one is a member of the HD2 family (HDT) [40]. HPTMs can regulate gene expression by changing chromatin structure, specifically influencing the tightness with which the nucleosomes bind to DNA and thereby shaping either a tight or loose chromatin state [41]. Different modifications can perform different functions, either promoting the packaging or the unpackaging of chromatin structure, leading to gene silencing or gene transcription, respectively (Figure 4). For instance, histone acetylation is associated with transcriptional activation, since the addition of acetyl groups to histone tails leads to weak binding of histones to DNA [42]. Histone methylation, on the other hand, can either activate or repress gene transcription, depending on the sites of methylation and the number of methyl groups added (me1, me2, me3) [39].

## 3. Epigenetic Changes Due to Adverse Environmental Conditions

Phenotypic plasticity constitutes an adaptation strategy that enables perennial plants to correspond efficiently to different environmental conditions [43]. Grapevine is considered one of the most highly plastic crops with an ability to cope with environmental heterogeneity. Accumulating data suggest that epigenetically driven phenotypic diversity enables grapevine to efficiently adjust to environmental changes, as implied by several plastic genes identified thus far (Table 1).

A recent study on three *V. vinifera* ‘Malbec’ clones (MB01, MB04, and MB10) cultivated in two different vineyards in Argentina with contrasting environmental conditions clearly showed that epigenetic diversity is a key contributor to inter-clonal variability [50]. The results showed that all clones had obvious phenotypic differences between vineyards, but no correlation between genetic and phenotypic variability was found. Instead, clone-dependent responses and a significant correlation between the environmentally induced epigenetic and phenotypic variations were detected, essentially demonstrating that DNA methylation plays a key role in phenotypic plasticity. The epigenetic variation observed was also influenced by microclimatic differences between vineyards, suggesting that the grapevine epigenome might contribute to the vineyard terroir.

Baránková et al. [51] using Merlot and Pinot Noir vines identified significant DNA methylation variability (79.9% and 70.7%, respectively), which was directly associated with the geographical location of vineyards (Czech Republic and Armenia). It is worth noting that variation in DNA methylation within the same vineyard represented only 14% (Pinot Noir group) or 16% (Merlot group) of the total level of variability recorded for each cultivar. The effects of different geographic locations on DNA methylation variability are better understood when considering the climatic conditions prevailing in different regions. Similarly, Xie et al. [52] showed a high level of differentiation among vineyards at the Barossa Valley in South Australia planted with cv. ‘Shiraz’, which could be explained by the distinct epigenetic profiles recorded rather than the low overall genetic variation. However, the separation between subregions was stronger for DNA methylation than for gene expression, suggesting that environmental specificities in each subregion influenced DNA methylation to a greater extent [44]. 

Methylation-sensitive genotyping-by-sequencing identified 3598 differentially methylated genes (DMGs) in grapevine leaves, 8.6% of which were associated with responses to abiotic factors, suggesting that environmental differences between locations probably contributed to the observed epigenetic variation [44,52,53]. The main contributors to the observed variations in DNA methylation patterns were the plant age and the average annual rainfall, the latter associated with the geographical location [44]. Several other studies have also showed that the grapevine age may be associated with variations in DNA methylation, which increases with the age of the vine [51,54,55,56,57]. The average annual rainfall data showed quite significant differences between the subregions, exposing vineyards to different irrigation regimes (from no irrigation to 1.2 ML/ha), associated with changes in DNA methylation and gene expression. Similar findings were also reported in other grapevine cultivars, such as ‘Chambourcin’ [43] and ‘Malbec’ [50,51,52,53,54,55,56,57,58], where water deficit conditions triggered DNA hypermethylation, while in the Italian variety ‘Bosco’, miRNA regulations were detected in response to drought stress [59].

Temperature also seems to be associated with changes in DNA methylation and gene expression patterns. Fabres et al. [44], by comparing subregions, revealed that those with the highest temperatures exhibited the most DMGs, indicating that Shiraz grapevines grown in warmer conditions accumulated more differences in DNA methylation than those from other subregions. In addition to high temperatures, low temperatures also seem to induce epigenetic changes in vine as demonstrated in a recent study with *V. amurensis* exposed to short- and long-term cold conditions. In particular, short-term chilling treatment resulted in 2793 increases and 305 decreases in H3K27me3 modification, most of which returned to their initial levels following extended exposure, suggesting a rapid epigenetic response to cold stress [46]. These results were also confirmed by Sun et al. [49] in *V. vinifera* cv. ‘Muscat Hamburg’, where the authors identified about 200 miRNAs responsible for targeting cold-responsive genes (*MYB*, *bHLH*, and *bZIP*), amongst which 44 were differentially expressed during chilling stress. 

Most epigenetic studies in grapevine have dealt with a single abiotic stressor (e.g., heat or drought), even though stress conditions in the environment usually occur in combination [14,53]. A recent study conducted with *V. vinifera* ‘Cabernet Sauvignon’ cv. revealed that an interactive effect of high temperature and drought stress on grapevine epigenome resulted in more DEGs under combined stress conditions compared to either stress individually [45]. Altitude was also shown to be correlated with the degree of DNA methylation in vines. Although higher altitude may be correlated with various factors, such as decreased temperature, increased precipitation, or increased sunlight intensity, changes in DNA methylation were rather attributed to increased solar UV radiation. In fact, altitude appears to have a direct effect on the levels of UV radiation experienced by plants. Relevant studies in different *V. vinifera* cultivars, i.e., ‘Shiraz’ [60], ‘Malbec’ [50,58,61], and ‘Tempranillo’ [62], and also in *V. amurensis* [63] demonstrated that UV-A, UV-B, and UV-C radiations can induce hypermethylation in the vine genome.

## 4. Epigenetic Effects on Berry Development and Quality

Grapevine berries exhibit notable phenotypic plasticity, with considerable variability observed within the same clone across different vineyards, as well as between berries of the same cluster or between clusters of the same vine [64]. Phenotypic plasticity is inextricably linked with epigenetic variation in grapevine berries, as evidenced in several epigenetically regulated genes involved in berry development and the biosynthesis of important metabolites (Table 2). For instance, Varela et al. [50] studied ‘Malbec’ clones cultivated in two distinct vineyards (Agrelo and Gualtallary in Mendoza, Argentina). The increased amount of total soluble solids, including sugars, observed in Agrelo was related to differentially methylated regions (DMRs) associated with genes involved in brassinosteroid homeostasis and activity. These genes play a role in controlling sugar partitioning in grapes [65,66]. The results showed several DMRs, mainly at CpG regions, corresponding to transcription factors and proteins with regulatory roles, such as E3 ubiquitin protein ligase, pentatricopeptide repeat proteins, and F-box proteins. 

Studies on Malbec grapevines revealed stress-induced DNA methylation changes in response to UV-B and abscisic acid (ABA) treatments. In particular, ABA application and increased UV-B radiation led to increased biosynthesis of low molecular weight polyphenols (LMWP) in berries and increased hydroxycinnamic acids (ferulic and caffeic acids) in young shoots [58]. Previous studies have also shown that high UV radiation can stimulate synthesis of non-flavonoid phenols, such as resveratrol, due to the upregulation of the stilbene synthase 10 (*VaSTS10*) gene [60,71]. This gene has been found to be regulated by various cytosine methylation patterns in protein-coding regions [56,60,72]. This was clearly shown in *V. amurensis* cell cultures by Kiselev et al. [73], where treatment with the demethylating agent 5-azacytidine exhibited reduced methylation levels of *VaSTS10*, whereas both gene expression and resveratrol synthesis were significantly increased, indicating that DNA methylation is involved in the regulation of resveratrol synthesis. 

**Figure 5:**
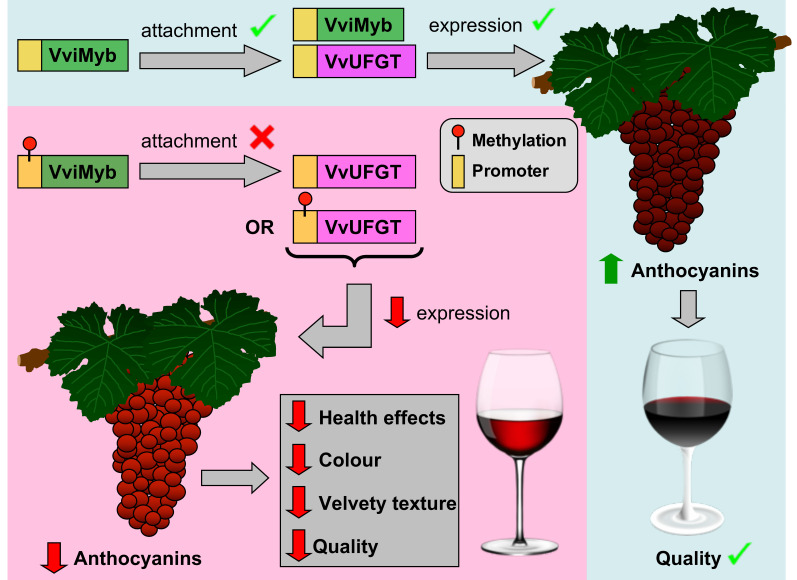
Effects of DNA methylation on anthocyanin content in grape berries and the resulting wine quality. Briefly, the binding of VViMyb transcription factors to the promoter region of *VvUFGT*, the key enzyme in anthocyanin biosynthesis, enhances its expression, resulting in elevated anthocyanin levels in grape berries. Conversely, methylation of either *VviMyb* or *VvUFGT* represses the expression of *VvUFGT* gene, resulting in poorly colored berries and diminished grape/wine quality.

DNA methylation has also been reported to affect wine quality by regulating the production of anthocyanins, a group of important phenolic metabolites in red wines contributing to wine color and also exhibiting beneficial health effects [47]. The color of berries in red and black grapevine cultivars is determined by the VviMybA1 and VviMybA2 transcription factors (TFs), which regulate the expression of *VvUFGT* (flavonol 3-O-D glucosyltransferase) that catalyzes the conversion of colorless anthocyanidin precursors to red and blue color anthocyanins (Figure 5). Studies have linked higher methylation levels of the *VviMyb* promoters to reduced *VvUFGT* expression [74,75]. Likewise, the methylation of the *VvUFGT* promoter has similar effects, as recently demonstrated in the study of Kong et al. [67]. In their investigation, cell suspensions derived from *V. vinifera* L. cv. ‘Gamay Teinturier’ were treated with the DNA methyltransferase inhibitor zebularine to explore the possible role of DNA methylation in the regulation of anthocyanin biosynthesis. Indeed, the results revealed an increased anthocyanin content in zebularine treatments, which is associated with reduced methylation levels of the *UFGT* promoter.

Some recent epigenetic studies have further suggested a role of histone post-translational modifications in grape berries [23]. Specifically, more than 30 genes encoding polycomb repressive complex 2 (PRC2) components (chromatin regulatory complex), SET domain group (SDG) proteins (epigenetic modulators with methyltransferase activity), and HATs were identified to exhibit expression patterns that indicate a possible involvement of the respective proteins in berry development and ripening [15,68]. Transcription factors of the NAC and MADS-box families also appear to be involved in grape berry ripening process. In contrast to grapevine leaves where several H3K27me3 marks were detected at the corresponding genes, in grapevine berries, the repressive H3K27me3 marks were removed and the genes were activated [69]. 

MicroRNAs (miRNAs), as epigenetic modulators, also appear to play a role in the ripening process of grape berries by controlling the secondary metabolism, e.g., by promoting anthocyanin and flavonol accumulation [70,76]. Finally, epigenetic modifications can affect berry composition also indirectly through alternative splicing (AS). Jia et al. [47] showed that the level of DNA methylation modified the AS of *VvDFR* (dihydroflavonol-4-reductase), *VvCHS* (chalcone synthase), and *VvGST* (glutathione S-transferase) genes by intron retention, altering the anthocyanin content in Kyoho berries during ripening. Further research is anticipated to contribute to a better understanding of the role that epigenetic regulation plays in modulating the secondary metabolism of grape berries under a variety of environmental conditions. This knowledge would be of particular importance for winegrowing to maintain high quality fruit and wine production in the view of global warming.

Besides grapes, epigenetics may have a global influence on the grape–wine ecosystem. The environmental conditions during fermentation, in particular, such as nutrient availability and temperature, can influence the epigenetic landscape of yeasts. Recently, Kong et al. [67], by using certain dietary compounds, reported for the first time a non-GMO method to alter the fermentation process of wines through epigenetic altering yeast gene transcription. Thus, understanding the role of epigenetics in various components of the grape–wine system could help in choosing correct viticultural practices and ensure the sustainability in wine industry.

## 5. Effects of Grafting on Grapevine Epigenome

Grafting is a major technique of asexual plant propagation, wherein an aboveground part of a plant (scion) is joined to the underground part (rootstock) of another. Rootstocks can originate from the same individual (self-grafting), different individuals of the same genotype (homografting), or distinct genotypes (heterografting). Grafting was initially used in viticulture to control phylloxera infection, an insect that was brought to Europe from America in the mid-19th century and eventually ruined most vineyards [77]. In addition to phylloxera resistance, grafting onto carefully selected rootstocks has been demonstrated to confer abiotic stress tolerance (e.g., drought, salinity) and improved resistance to pests and diseases [78]. The interplay between the rootstock and environmental factors may have significant effects on the formation of vine scion’s phenotype, thus having an impact on wine terroir [64,79,80]. Considering that phenotypic diversity is linked to epigenetic variation, several intriguing questions emerge: Do different rootstocks generate distinct epigenotypes? Can epigenetic changes lead to predictable scion phenotypes? Is phenotypic stability linked to epigenotypes derived from specific scion–rootstock pairings? How do rootstock effects on scion’s epigenome vary with environmental conditions? 

A study with clonal replicates of cv. ‘Chambourcin’ showed that the methylome was sensitive to both irrigation and grafting. Although ungrafted vines exhibited high epigenetic variation under different irrigation regimes, grafting contributed to more stable DNA methylation patterns [43]. This may suggest additional viticultural benefits of grafting, such as stability of grapevine performance for important agricultural traits. Recent research showed that the epigenetic crosstalk between rootstock and scion involves the transportation of sRNAs between grafting partners [15]. In *V. vinifera* cv. ‘Riesling’ grafted onto rootstock ‘C3309′, about 13% of the total protein coding genes, including genes responsible to abiotic stress responses and signal transportation, were found to produce mobile mRNAs across graft junctions [81]. It is worth noting that even though this communication is bidirectional, it exhibits a preferential scion to rootstock movement, perhaps due to a source–sink flow [82]. Rubio et al. [82] investigated sRNA populations in one homograft (‘Cabernet Sauvignon’ cv.) and two different heterografts. They discovered that endogenous repeated sequences located in the scion induce DNA methylation in the rootstock by producing mobile siRNAs. Interestingly, the targets of these siRNAs were found to be more methylated in heterografts compared to homografts. Harris et al. [14] compared ungrafted grapevines with three heterografted of the same scion (cv. ‘Chambourcin’) in order to examine the effects of grafting on scion’s gene expression. The results showed no DEGs between heterografts, but significant differences were detected compared to the ungrafted vines, indicating that the observed gene expression diversity is a result of heterografting per se and does not derive from specific genotype-to-genotype interactions. Similarly, heterografting changed gene expression in ‘Cabernet Sauvignon’ tissues, irrespective of the rootstock genotype [83]. However, in another study [84], variations in cv. ‘Gaglioppo’ leaves seemed to be influenced by the genotype of the rootstock, suggesting that besides the type of grafting (homograft or heterograft), which significantly affects sRNA populations regardless of the rootstock genotype, there may also be genotype-specific effects. In support of this, a specific rootstock-to-genotype effect has been reported [82], indicating a distinct influence of each particular rootstock on scion’s smRNA population.

## 6. Epigenetics and Defense Responses to Pathogens

The last decade’s studies have shown that pathogen infection may result in epigenetic modifications that promote plant defenses in local and systemic tissues and are often inherited by offspring [85]. The role of DNA methylation in plant defense responses was initially described in resistance against DNA viruses by mediating transcriptional gene silencing in viral genomes. Recent studies provide further evidence that this mechanism also modulates immune responses against other pathogens as well [86]. Most of the research on this topic suggests that reduced DNA methylation increases the responsiveness of the plant immune system [12]. Indeed, in grapevine, the resistance conferred to leafroll-associated virus 3 (GLRaV-3) in transgenic plants expressing the coat protein GLRaV-3 was negatively correlated with the overall levels of genome methylation [87].

Hypomethylated Arabidopsis mutants displayed enhanced resistance to *Hyaloperonospora arabidopsidis*, a pathogen with similar biology to *Plasmopara viticola*, the causal agent of the downy mildew of grapevine. On the contrary, two hypermethylated Arabidopsis mutants were more susceptible to the same pathogen. Subsequent characterization of the hypomethylated *nrpe1* mutant, which is impaired in RNA-directed DNA methylation, and the hypermethylated *ros1* mutant, which is affected in DNA demethylation, revealed opposite phenotypes with the resistant phenotype expressing cell wall defenses and salicylic acid-dependent gene expression [88]. Likewise, a study in grapevines reported that during the incompatible interaction of the tolerant grapevine cultivar ‘Regent’ with *P. viticola*, *DNMTs* and *CMTs* were downregulated within six hours post-infection, leading to global cytosin hypomethylation. In contrast, the compatible interaction was characterized by hypermethylation at the same time point [25]. Similarly, Azevedo et al. [89] reported that a grapevine genotype tolerant to *P. viticola* exhibited lower methylation levels compared to a susceptible genotype and displayed an early enhanced expression of defense- and epigenetics-related genes upon infection. Another study, though, in Arabidopsis revealed that transgenerational systemic acquired resistance against *H. arabidopsidis* failed in mutants impaired in RNA-directed DNA methylation (RdDM) [90]. This finding contradicts the previously mentioned studies, yet it implies the significant role of RdDM in plant response to pathogens.

A transcriptome analysis of *V. pseudoreticulata* (Chinese wild grapevine) infected with the powdery mildew pathogen *Erysiphe necator* aimed to provide insights into grapevine resistance mechanisms. The analysis revealed that many DEGs identified were related to defense responses. Interestingly, the host response also involved the downregulation of genes involved in methylation [91]. In an attempt to study the role of histone methyltransferase genes (HMs) in grapevine response against *E*. *necator*, Wang et al. [92] studied their expression profile at 12 and 24 h post-inoculation (hpi). At the earliest time point, the expression of three HM genes (*VvHAC1*, *VvHAG4*, and *VvHAG23*) was significantly upregulated but subsequently downregulated at 24 hpi. On the contrary, the expression of six other HM genes (*VvHAM2*, *VvHDA1*, *VvPRMT4*, *VvHDT1*, *VvSDG38*, and *VvSRT2*) was downregulated at 12 hpi and upregulated at 24 hpi. Their results collectively suggest that some of the *V. vinifera* HM genes are responsive to powdery mildew and, therefore, might have a role in pathogen resistance.

A role of DNA methylation in grapevine’s interaction with the necrotrophic pathogen *B. cinerea* has also been suggested. Transcriptomics in grapevine berries revealed that genes involved in epigenetic modifications, such as DNA (cytosine-5)-methyltransferase, helicases, DICER and ARGONAUTE proteins, were differentially expressed upon Botrytis infection [93]. Nerva et al. [94] showed that spray-induced gene silencing (SIGS) using double-stranded RNA (dsRNA) can induce plant resistance against *B*. *cinerea* in ‘Moscato’ cv. grafted onto Kober 5BB rootstock. The role of dsRNAs in RNA-directed DNA methylation has previously been well described [95]. In the above studies, though, the DNA methylation levels were not assessed and there is no clue to how they might affect plant response against the pathogen. In Arabidopsis, the development of crown gall tumors caused by the soilborne biotrophic pathogen *Agrobacterium tumefaciens*, one of the *Agrobacterium* species causing crown gall disease in grapevine, was suppressed by DNA methylation [86]. Compared to the wild-type, mutants with lower non-CG methylation generated larger tumors, indicating that hypermethylation in *A. tumefaciens* slows the formation of plant tumors, in contrast to other pathogen infections [96]. Nevertheless, there has been no study yet confirming such an effect in grapevine.

## 7. Epigenetic Memory

The priming phenomenon constitutes an adaptive strategy in which plants “memorize” stressful events, partly memorized through epigenetics, to more efficiently cope with similar conditions in the future [51,97]. In the grapevine cvs. ‘Asgari’ (drought-tolerant) and ‘Yaghooti’ (drought-sensitive), drought stress priming increased cold tolerance in shoots and roots. Similarly, Pagay et al. [98] observed increased drought tolerance in non-irrigated ‘Cabernet Sauvignon’ grapevines, accompanied by improved water status, leaf gas exchange values, and berry size, which appeared to be associated with long-term adaptation to drought stress through priming. Interestingly, grapevines of cv. ‘Schioppettino’ infected with grapevine leaf spot virus (GFLV) exhibited greater resistance to mild water stress than healthy vines [99], suggesting that biotic stress could potentially trigger abiotic stress priming in grapevine. In addition to the above examples, spray-induced gene silencing (SIGS) targeting a glutathione transferase gene (*VvGST40*) was demonstrated to increase grapevine drought tolerance through priming mechanisms [100]. Furthermore, 6-Benzylaminopurine (BAP) promotes salt tolerance, with BAP-primed grapevines exhibiting higher water use efficiency, PSII efficiency, and growth rate [101]. 

**Figure 6:**
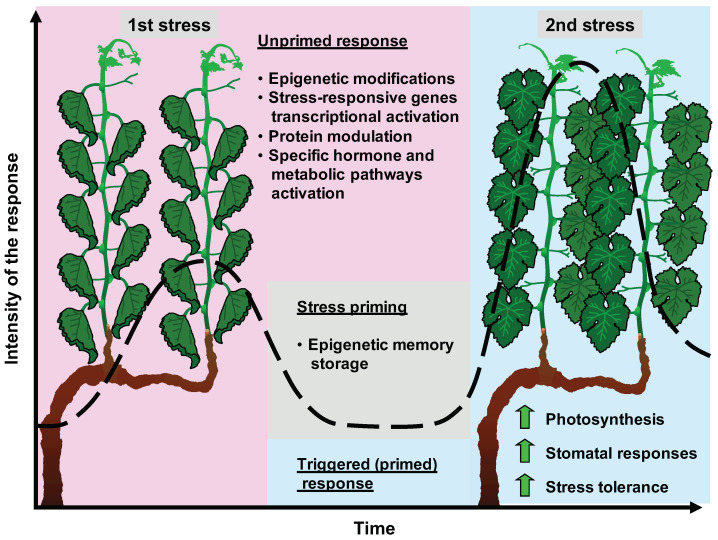
Epigenetic memory effects on grapevine transcriptional and physiological responses to repeated stress conditions. Briefly, when a stressor is encounter for the first time, it triggers a so-called unprimed response. It involves activation of stress response mechanisms, including epigenetic modifications, that impact the expression of stress-related genes. These modifications allow for cells to establish epigenetic memory that enhances their ability to respond to subsequent stimuli, leading to improved stress tolerance.

Following a stress, when the plant enters in a primed state, most stress-responsive genes revert to their original expression levels. However, some stress-inducible genes do not revert to the previous epigenetic state, thereby contributing to the establishment of the so-called epigenetic memory. Epigenetic memory is maintained during the recovery period and becomes reactivated upon encountering a similar stress [15]. Recent studies have started to reveal a prominent role of epigenetic memory in grapevine responses to recurring stress conditions (Figure 6). In grapevine, after the termination of thermotherapy-imposed stress, alterations in DNA methylation gradually returned to the previous levels, while regenerants returned to epigenetic states similar to those of the maternal plants, 6 weeks to 3 years later. Specifically, 40% of the observed diversity disappeared within a year of stress termination, reflecting transient changes, whereas 60% of the DNA methylation changes remained more than a year, probably reflecting the establishment of long-term stress memory [56]. In ‘Cabernet Sauvignon’, investigating the epigenetic effects of drought, heat, and combined stress, a small number of DEGs remained after heat and combined stress, but no DEGs remained after drought stress [45].

In clonally propagated plants like grapevines, where epigenetic marks transmission occurs through mitosis, there is clear evidence that epigenetic marks may inherited from parents to asexually multiplied offspring, possibly reflecting the parents’ environmental adaption [15,102]. Supporting this, DNA methylation analysis from grapevines grown in different vineyards across Argentina showed a stronger correlation with clonal origin rather than geographical location [50]. These data suggest that transgenerational stress memory may serve as an innovative strategy for improving grapevine adaptation to climate change [15]. 

Priming is also a component of the so-called inducible plant defense [103,104,105]. When a plant encounters a pathogen attack, it often becomes more resistant to subsequent pathogen infections, a phenomenon known as systemic acquired resistance (SAR) [106]. Non-pathogenic root-colonizing microbes can also prime the immune system to enhance defenses that are only activated upon pathogen infection. This phenomenon is known as induced systemic resistance (ISR) [107,108]. A role of epigenetic regulation in the defense priming has been demonstrated in a number of studies [105,106,107,108,109]. For instance, in Arabidopsis, chromatin modification has been shown to act as a memory for SAR [110]. Additionally, a variety of chemicals can imitate biologically induced priming events. When these compounds are applied, the priming response becomes less varied and more constant. The majority of the priming-inducing chemicals are endogenous plant compounds or functional analogues synthesized by the plant in response pathogen attack. Examples include salicylic acid (SA) [111], jasmonic acid (JA) [112], azelaic acid [113], and beta-aminobutyric acid (BABA) [114], with the latter being effective in potentiating defense responses in grapevine against downy mildew. 

## 8. Conclusions

In years to come, it is expected that grape growers will face new challenges due to climate change and the possible subsequent rising of pest and diseases [115]. The effects of extremely hot temperatures and drought for prolonged periods in warmer climate zones is already obvious in the winegrowing of many countries [5,116]. However, grapevines have the ability to adapt in a changing environment due to phenotypic plasticity, a common phenomenon in vines that is often mediated by epigenetic modifications [117]. 

Although research on grape epigenomics is still in early stages, compelling recent evidence has documented that epigenetic regulatory mechanisms are involved in many aspects of grapevine development and also in grape adaptation to variable and often harsh environmental conditions. Research on the role of environmental conditions on the grapevine epigenome indicates that several environmental constrains, such as water availability, extreme temperatures, and UV radiation, may create phenotypic variation by altering the transcriptome through epigenetic changes [43,44,46,63]. Such studies have mainly focused on DNA methylation and a single stress factor. Nevertheless, since heat stress combined with drought stress have been shown to have an additive effect on DEGs [45], it is important to further investigate the role of combined stresses on the grapevine epigenome. A few studies have also revealed the existence of epigenetic crosstalk between rootstock and scion, indicating that grafting can influence epigenetic modifications, especially DNA methylation. These modifications likely impact vine performance in response to stress [83,84]. 

Despite the paucity of published studies, it seems that epigenetics is an important component in grapevine defenses against pathogens. Enhancing our understanding of the epigenetic regulation of plant immunity may result in novel tools to strengthen natural defenses and thereby manage grapevine’s diseases and pests in an eco-friendly manner. Viticulture can also greatly benefit from understanding the mechanisms of transient and stable modifications in epigenetic memory for the development of new epi-breeding techniques for stress adaptation, such as targeted epigenetic modifications that can lead to more stress-tolerant cultivars [53].

Winemakers carefully consider grapevine phenotypic traits when making decisions about vineyard management, grape harvest, and winemaking processes to achieve the desired wine quality. As far as wine quality is driven by the grape phenotype, which is epigenetically shaped by environmental factors, regional-specific epigenetic regulation of important metabolites in grape berries might shape wine characteristics, thus potentially contributing to the so-called wine terroir concept. Phenotypic plasticity of grape berry traits has linked epigenetic variations with changes in skin dry weight, total soluble solids, resveratrol concentration, anthocyanin and flavonoid biosynthesis [50,70,73,76]. In view of climate change, critical exploration into the impact of epigenetic modifications on key enological traits is imperative to uphold the quality standards of wine production. 

## Figures and Tables

**Table 1:** List of plastic grapevine genes/proteins that are epigenetically regulated under abiotic stress conditions.

Genes/Proteins	Stress or Function	Epigenetic Process	Organ	*V. vinifera* cv./ *Vitis* sp.	Geographic Location	Reference
C2-domain ABA-related (CAR) proteins	Drought, ABA pathway	DNA methylation	Berries	Shiraz	Barossa Valley, Australia	[44]
Histone-lysine methyltransferase-related genes (e.g., *SUV3*)	Heat-drought	Histone modification	Leaves	Cabernet Sauvignon	South Australia	[45]
Chitinase-related genes	Cold stress	H3K27me3	Leaves	*V. amurensis*	Jilin, China	[46]
G-type lectin s-receptor-like serine/threonine-protein kinase	Cold stress	H3K27me3	Leaves	*V. amurensis*	Jilin, China	[46]
Glucosyltransferases (GTFs)	Cold stress	H3K27me3	Leaves	*V. amurensis*	Jilin, China	[46]
Peroxidases (PODs)	Cold stress	H3K27me3	Leaves	*V. amurensis*	Jilin, China	[46]
NAC domain (NAM, ATAF1/2 and CUC2)	Cold stress	H3K27me3	Leaves	*V. amurensis*	Jilin, China	[46]
Ethylene-insensitive 3 (*EIN3*) transcription factor	Dormancy/ bud break	DNA methylation	Buds	Kyoho	Nanjing, China	[47]
Gibberellin-related (*GA*) genes	Dormancy/ bud break	DNA demethylation	Buds	Kyoho	Nanjing, China	[47]
WRKY domain transcription factors	Cold stress	H3K27me3/DNA demethylation	Leaves	Fleurtai, UD 31-103	Northern Italy	[47]
DEMETER-like DNA demethylase genes (*VvDEM1*, *VvDEM2*, *VvDEM3*)	Dormancy/bud break	DNA demethylation	Buds	Kyoho, *V. amurensis*	China	[48]
*MYB* (myeloblastosis) domain transcription factors	Cold/drought	miRNAs/DNA demethylation	Leaves	Kyoho, Muscat Hamburg	China	[47,49]
bHLH (basic helix–loop–helix) domain transcription factors	Cold/drought/ ABA pathway	miRNAs/DNA demethylation	Leaves	Kyoho, Muscat Hamburg	Beijing, China	[47,49]
bZIP (basic-leucine zipper) domain transcription factors	Cold stress	miRNAs	Leaves	Muscat Hamburg	Beijing, China	[49]
AP2/ERF (APETALA2/ Ethylene-responsive) transcription factors	Cold stress	miRNAs	Leaves	Muscat Hamburg	Beijing, China	[49]
SBP (SQUAMOSA PROMOTER BINDING PROTEIN) transcription factors	Cold stress	miRNAs	Leaves	Muscat Hamburg	Beijing, China	[49]

**Table 2:** List of grapevine plastic genes involved in berry development and ripening, whose expression is epigenetically regulated.

Genes/Proteins	Function	Epigenetic Process	*V. vinifera* cv./ *Vitis* sp.	Geographic Location	Reference
E3 ubiquitin protein ligases	Development–ripening	DNA methylation	Malbec	Gualtallary, Argentina	[58]
Pentatricopeptide repeat proteins	Development–ripening	DNA methylation	Malbec	Mendoza, Argentina	[50]
F-box protein domain encoding genes	Development–ripening	DNA methylation	Malbec	Mendoza, Argentina	[50]
Oxygenase encoding gene (VIT_15s0048g01960)	Sugar content control	DNA methylation	Malbec	Mendoza, Argentina	[50]
Oxysterol-binding protein-related protein 4B-like encoding gene (VIT_11s0103g00530)	Sugar content control	DNA methylation	Malbec	Mendoza, Argentina	[50]
O-acyltransferase (WSD1-LIKE) gene family	Berry skin dry weight control	DNA methylation	Malbec	Mendoza, Argentina	[50]
AP2/ERF (APETALA2/ Ethylene-responsive factor) transcription factors	Berry skin dry weight control	DNA methylation	Malbec	Mendoza, Argentina	[50]
Stilbene synthase *VaSTS10*	Resveratrol biosynthesis	DNA methylation	Shiraz	Barossa Valley, Australia	[60]
UDP-glucose-flavonoid 3-O-glucosyltransferase *VvUFGT*	Anthocyanin biosynthesis	DNA methylation	Gamay Teinturier	Bordeaux, France	[67]
SET DOMAIN GROUP (SDG) proteins	Flowering/ grape development	H3K27me3	Cabernet Sauvignon	Central Chile	[68]
No apical meristem (*NAM*) gene	Ripening control	H3K27me3	Pinot noir	Hong Kong, China	[69]
MADS-box transcription factors	Ripening control	H3K27me3	Pinot noir	Hong Kong, China	[69]
*VvMYBA1*, *VvMYBA2*	Anthocyanin biosynthesis	DNA methylation	Gamay Teinturier	BarossaValley, Australia	[67]
*VvMYB114*	Flavonoid biosynthesis	miRNAs	Dilkhush, Bangalore Blue, Red Globe	Bengaluru, India	[70]
VvO-methyltransferase 3 (VvOMT3)	Methoxypyrazines (MPs) biosynthesis	H3K27me3	*V. amurensis*	Jilin, China	[46]
Dihydroflavonol reductase *VvDFR*	Anthocyanin accumulation	DNA methylation	Kyoho	Nanjing, China	[47]
Glutathione S-transferase *VvGST*	Anthocyanin accumulation	DNA methylation	Kyoho	Nanjing, China	[47]
chalcone synthase *VvCHS*	Anthocyanin accumulation	DNA methylation	Kyoho	Nanjing, China	[47]
